# A Tear Metabolomic Profile Showing Increased Ornithine Decarboxylase Activity and Spermine Synthesis in Thyroid-Associated Orbitopathy

**DOI:** 10.3390/jcm11020404

**Published:** 2022-01-13

**Authors:** Benjamin Billiet, Juan Manuel Chao de la Barca, Marc Ferré, Jeanne Muller, Anaïs Vautier, Sophie Assad, Odile Blanchet, Lydie Tessier, Céline Wetterwald, Justine Faure, Geoffrey Urbanski, Gilles Simard, Delphine Mirebeau-Prunier, Patrice Rodien, Philippe Gohier, Pascal Reynier

**Affiliations:** 1 Département d’Ophtalmologie, Centre Hospitalier Universitaire (CHU), F-49000 Angers, France; benjamin.billiet.chu@gmail.com (B.B.); jeanne.muller@chu-angers.fr (J.M.); vautier.anais@gmail.com (A.V.); sophie.assad@chu-angers.fr (S.A.); PhGohier@chu-angers.fr (P.G.); 2 Service de Biochimie et Biologie Moléculaire, Centre Hospitalier Universitaire (CHU), F-49000 Angers, France; jmchaodelabarca@chu-angers.fr (J.M.C.d.l.B.); lytessier@chu-angers.fr (L.T.); cewetterwald@chu-angers.fr (C.W.); justine.faure@chu-angers.fr (J.F.); gisimard@chu-angers.fr (G.S.); deprunier@chu-angers.fr (D.M.-P.); 3 Unité Mixte de Recherche (UMR) MITOVASC, Centre National de la Recherche Scientifique (CNRS), Institut National de la Santé et de la Recherche Médicale (INSERM), Université d’Angers, F-49000 Angers, France; marc.ferre@univ-angers.fr (M.F.); geoffrey.urbanski@chu-angers.fr (G.U.); parodien@chu-angers.fr (P.R.); 4 Centre de Ressources Biologiques, BB-0033-00038, Centre Hospitalier Universitaire (CHU), F-49000 Angers, France; odblanchet@chu-angers.fr; 5 Service de Médecine Interne et d’Immunologie Clinique, Centre Hospitalier Universitaire (CHU), F-49000 Angers, France; 6 Service d’Endocrinologie, Centre Hospitalier Universitaire (CHU), F-49000 Angers, France

**Keywords:** Graves’ ophthalmopathy, metabolomics, ornithine decarboxylase, spermine, thyroid-associated orbitopathy

## Abstract

About half of patients with Graves’ disease develop an orbitopathy related to an inflammatory expansion of the periorbital adipose tissue and muscles. We used a targeted metabolomic approach measuring 188 metabolites by mass spectrometry to compare the metabolic composition of tears in patients with active (*n* = 21) versus inactive (*n* = 24) thyroid-associated orbitopathy. Among the 44 metabolites accurately measured, 8 showed a significant alteration of their concentrations between the two groups. Two short-chain acylcarnitines, propionylcarnitine and butyrylcarnitine, and spermine showed increased concentrations in the tears of patients with active orbitopathy, whereas ornithine, glycine, serine, citrulline and histidine showed decreased concentrations in this group. In addition, the ratio putrescine/ornithine, representing the activity of ornithine decarboxylase, was significantly increased in patients with active compared to inactive orbitopathy (*p* = 0.0011, fold change 3.75). The specificity of this candidate biomarker was maintained when compared to a control group with unclassified dry eye disease. Our results suggest that the stimulation of ornithine decarboxylase by TSH receptor autoantibodies in orbital fibroblasts could lead to increased synthesis of spermine, through the increased activity of ornithine decarboxylase, that may contribute to periorbital expansion in Graves’ ophthalmopathy.

## 1. Introduction

Thyroid-associated orbitopathy (TAO), or Graves’ ophthalmopathy, has an estimated prevalence of 1/1000 in Europe [1]. Women between 40 and 50 years are more frequently affected in accordance with the epidemiology of Graves’ disease itself. Hyperthyroidism in Graves’ disease results from an over-stimulation of the thyroid cells by TSH receptor autoantibodies, resulting in the increased secretion of thyroid hormones [2]. TAO is also directly caused by these autoantibodies, which stimulate the orbital fibroblasts also expressing the TSH receptor. The activated fibroblasts differentiate into myofibroblasts and adipocytes [3]. The myofibroblasts secrete glycosaminoglycans in oculomotor muscles, leading to their increased volume. In parallel, neoadipogenesis causes an expansion of the fat volume in the orbit. Activated orbital fibroblasts also produce inflammatory mediators that facilitate the orbital trafficking of monocytes and macrophages, promote differentiation of matrix-producing myofibroblasts and stimulate the accumulation of a hyaluronan-rich stroma, leading to orbital tissue edema and fibrosis [4].

Approximately half of patients with Graves’ disease develop an orbitopathy, which most often occurs in the months before or after the onset of dysthyroidism [5]. This orbitopathy can be manifested by a dry and gritty ocular sensation, photophobia, sensation of excessive tearing, diplopia and a sensation of pressure behind the eyes [2]. On clinical examination, proptosis, restricted ocular motility, eyelid retraction, palpebral edema and erythema of the periorbital tissues may be present. TAO can be complicated by severe pain, corneal ulceration, or compressive optic neuropathy, with a risk of vision loss. Smoking is a significant risk factor for TAO in patients with Graves’ disease [6].

The clinical management of TAO depends on its severity. The European Group on Graves’ Orbitopathy (EUGOGO) guidelines distinguish mild from moderate and severe orbitopathy [7]. The Clinical Activity Score (CAS), based on clinical signs of inflammation, also helps to differentiate active TAO needing treatment from inactive TAO [8]. The management of TAO is primarily based on the treatment of dysthyroidism, smoking cessation, and local symptomatic treatments. Treatment of dysthyroidism is based on synthetic antithyroid drugs and may be reinforced by surgery or radiotherapy. Severe forms of TAO require systemic corticosteroid therapy, sometimes followed by radiotherapy and even immunosuppressive drugs.

The search for tear biomarkers in ocular diseases has gained considerable attention with the advent and increased sensitivity of omics approaches [9]. Tears have a protective, nourishing and lubricating role on the ocular surface. They are composed of three layers: an outer lipid layer produced by the meibomian glands, an intermediate layer produced by the lacrimal glands with an aqueous composition and an inner layer produced by the goblet cells composed of mucus in contact with the epithelial surface [10]. Tears contain hundreds of compounds including ions, carbohydrates, lipids and proteins, as well as various metabolite families. To our knowledge, no metabolomic studies have been reported so far in tears of patients with TAO, in contrast to other causes of dry eye disease that have been much more well studied, such as in the Sjögren syndrome [11]. However, a blood metabolomic profiling in Graves’ disease identified ten discriminating metabolites, most of which showed a positive correlation with thyrotropin receptor antibodies [12]. In this study, only two blood metabolites—proline and 1,5-anhydroglucitol—were found to be discriminating in the presence of orbitopathy. In addition, in comparison with healthy controls, patients with orbitopathy showed a specific profile in the orbital adipose/connective tissues in which fumarate, proline, phenylalanine and glycerol were coordinately altered with the blood metabolites.

Here, we present a targeted quantitative metabolomics approach, comparing the metabolic composition of tears in patients with active versus inactive TAO.

## 2. Materials and Methods

### 2.1. Study Participants

The participants were included in the study after having given their informed written consent for the research. The study was conducted according to the ethical standards of the Helsinki Declaration and its later amendments, and with the approval of the ethical committee (Comité de Protection des Personnes, CPP-OUEST 2), agreement number CB 2016-08.

Individuals were prospectively recruited during routine consultation in the Department of Ophthalmology of the Angers University Hospital, France, from June 2019 to October 2020. All included patients were diagnosed with Graves’ ophthalmopathy by an ophthalmologist together with an endocrinologist. The patients were divided into two groups according to their Clinical Activity Score (CAS) [8]. Patients with a CAS greater than or equal to 3 were included in the “active TAO” group (*n* = 21) and those with a CAS less than 3 in the “inactive TAO” group (*n* = 24). They had their past ophthalmic history recorded and their clinical assessment was included. The CAS clinical parameters were collected (spontaneous retrobulbar pain, pain with upward or downward eye movements, redness of the eyelids, conjunctival hyperemia, palpebral edema, inflammation of the caruncle and/or semilunar fold and chemosis), as well as visual acuity, presence or absence of optic disc edema, tear film break-up time measurement, smoking activity, medical and ophthalmologic history and systemic and ophthalmologic medications were also recorded. Previous treatments for severe Graves’ disease were also recorded, including corticosteroids, radiotherapy and thyroidectomy. The paraclinical parameters collected were the Schirmer strip imbibition measurement in millimeters, TSH/T3/T4 and TSH receptor autoantibodies (TRAb) plasma levels. The exclusion criteria were other concomitant ophthalmic and thyroid diseases.

An independent control collection of tears from individuals (*n* = 20) with an objective eye dryness of unknown origin was further used to address the specificity of the putrescine/ornithine ratio. Eye dryness was quantified by an Oxford score, evaluating the severity of dry eye, higher than 1 and/or a Schirmer I test ≤ 5 mm/5 min and/or a break-up time (BUT) test < 10 s on at least one eye.

### 2.2. Tear Sampling and Metabolite Extraction

The Schirmer strip tears collection was performed in fasting patients in the morning using Schirmer-Plus strips (GECIS^®^, Neung sur Beuvron, France) in sterile conditions, placed at the 2-3/1-3 outer junction of the lower eyelid and left in place for 5 min, without prior local anesthetic. The tear-soaked strips were then placed in Eppendorf^®^ tubes, which were immediately transferred in liquid nitrogen, before being sent to our Biological Resource Center for conservation at −80 °C.

A tear volume of 15 µL was collected as described [11] by cutting the soaked strips at 21 mm from their edge. The strips were then transferred into precooled 2.0 mL homogenization Precellys tubes (Bertin Technologies, Montigny-le-Bretonneux, France) prefilled with 1.4-mm diameter ceramic beads and 20 μL of cold methanol. The strips were homogenized by two grinding cycles, each at 6500 rpm for 30 s, spaced 20 s apart, using a Precellys^®^ homogenizer kept at +4 °C. The supernatant containing the metabolites was recovered after centrifuging at 20,000× *g* for 10 min and kept at −80 °C until analysis.

### 2.3. Metabolite Analysis

Targeted quantitative metabolomics analysis was carried out using the Biocrates^®^ Absolute IDQ p180 kit (Biocrates Life Sciences AG, Innsbruck, Austria) coupled with a QTRAP 5500 (SCIEX, Villebon-sur-Yvette, France) mass spectrometer (MS), a 1260 Agilent^®^ High Performance Liquid Chromatography (HPLC) system and an ECLIPSE^®^ XDB-C18 3.5 μm 3.0 × 100 mm column (Agilent Technologies, Santa Clara, CA, USA) as previously described [11]. All reagents used in this analysis were of LC–MS grade and purchased from VWR (Fontenay-sous-Bois, France) and Merck (Molsheim, France). The metabolomics kit enables the quantification of up to 188 different endogenous molecules, including acylcarnitines (40), amino acids (21), biogenic amines (21), glycerophospholipids (90), sphingolipids (15) and hexoses (1). Flow-injection analysis (FIA–MS/MS) was used to quantify acylcarnitines, glycerophospholipids, sphingolipids and sugar, whereas liquid chromatography allowed us to separate amino acids and biogenic amines prior to detection with mass spectrometry (LC–MS/MS). The samples were prepared according to the Biocrates Kit User Manual. Briefly, extracts were vortexed thoroughly for 5 min, then 10 μL was mixed with isotope-labeled internal standards and the samples were loaded onto the center of the filter placed on the upper wall of the well in a 96-well plate. Metabolites were extracted in a methanol solution using ammonium acetate after drying the filter spot under a nitrogen flow and derivatizing with phenylisothiocyanate for the quantification of amino acids and biogenic amines. The extracts were diluted with MS running solvent (MilliQ water for HPLC assay or a methanol solution for FIA assay) prior to FIA and LC–MS/MS analyses. Peaks were integrated in order to retrieve raw data as a matrix compiling the concentrations of the 188 metabolites for the 80 samples, in addition to the calibration samples and quality control (QC) values. Three QCs composed of three concentrations of human samples, i.e., low (QC1), medium (QC2) and high (QC3), were used to evaluate the performance of the analytical assay. A seven-point serial dilution of calibrators was used to generate the calibration curves. The values of the coefficient of variation (CV = standard deviation/mean × 100, in %) associated with the QC samples were used to validate quantitation in the samples (CV threshold of 30%). The software Analyst (SCIEX, Villebon-sur-Yvette, France) was used for MS data collection and the software MetIDQ^®^ (Biocrates Life Sciences AG, Innsbruck, Austria) was used to monitor the entire assay workflow.

### 2.4. Statistical Analysis

After validation of the three levels of quality control used with the kit, the metabolite concentrations were only used for statistical analysis if they were in the quantitation range determined by the calibration curves. Indeed, metabolites with more than 20% of their values outside of the quantitation range (i.e., concentrations above the upper limit of quantitation or below the lower limit of quantitation) were not considered. Before excluding a given metabolite with more than 20% outside of the quantitation range from statistical analysis, a χ^2^ test was performed with the independence between in/out of range and active/inactive TAO properties as the null hypothesis.

A Student t test was used to compare the metabolite concentrations between the active and inactive TAO groups. A non-parametric Mann–Whitney–Wilcoxon test was applied for comparing the metabolite ratios between groups, whilst a Student t test was used for comparisons involving metabolite sums. *p*-values less than α = 0.05 were considered significant.

## 3. Results

### 3.1. Clinical Description

The demographic and clinical data of the two groups of patients included in this study are presented in Table 1. There were no differences in age, sex, morbidity and thyroid status between the two groups, except for a higher proportion of active smokers in the inactive CAT group. As expected, the Oxford score and the CAS were significantly different between the two groups.

### 3.2. Altered Metabolite Concentrations

From the 188 metabolites measured, 44 were found to be correctly measured. Supplementary Table S1 shows these 1980 concentrations (μM/L) of metabolites measured in the tears of the 45 individuals. Of these 44 metabolites, 8 were found to be significantly altered in the active compared to the inactive orbitopathy (Table 2). The polyamine spermine showed increased levels in active TAO (*p* = 0.0074, fold change 1.34), as well as two short-chain acylcarnitines, propionylcarnitine (C3, *p* = 0.0232, fold change 1.77) and butyrylcarnitine (C4, *p* = 0.0176, fold change 1.54). Five amino acids showed decreased concentrations: ornithine (*p* = 0.0189, fold change 0.43), glycine (*p* = 0.0235, fold change 0.45), serine (*p* = 0.0278, fold change 0.38), citrulline (*p* = 0.0281, fold change 0.23) and histidine (*p* = 0.0336, fold change 0.45). Finally, among the sums and ratios of metabolites with functional importance proposed by Biocrates, one was significantly modified: the ratio of putrescine to ornithine, which represents the activity of ornithine decarboxylase (ODC), showed an increased value in active TAO (*p* = 0.0011, fold change 3.75).

To address the specificity of the putrescine/ornithine ratio in the active form of TAO, an independent control group of tears from individuals (*n* = 20) with dry eye of undetermined origin was used. The demographic and clinical data of these control patients are presented in Table 3. The majority of these patients were included in this group on the basis of an altered Break Up Time test. The specificity of this increased ratio in active TAO was confirmed since it remained significantly increased in active TAO compared to this control group (*p* = 0.02) without significant difference (*p* = 0.12) when comparing inactive TAO with this control group.

## 4. Discussion

Our study reveals an alteration to eight metabolite concentrations and one enzymatic activity in the tears of patients with active compared to inactive TAO.

The increased concentrations of two short-chain acylcarnitines is interesting because the return to a euthyroid stage after three months of treatment of Graves’ disease has previously been associated with a decrease in blood concentrations of short-chain acylcarnitines [13]. Our study reveals that in tears, active and inactive forms of thyroid orbitopathy are also distinguished by altered concentrations of short-chain acylcarnitines. These metabolites are related to the catabolism of glucose and amino acids rather than the oxidation of fatty acids, as is the case with medium- and long-chain acylcarnitines. Therefore, the increase in periorbital short-chain acylcarnitines may reflect an increased oxidative catabolism of glucose and amino acids to meet the higher energy requirements of the activated fibroblasts in active TAO.

Ornithine decarboxylase (ODC) catalyzes the decarboxylation of ornithine, leading to the formation of putrescine. The putrescine/ornithine ratio, increased by 3.75 times in the tears of patients with active TAO, shows the increased activity of this enzyme in the ocular environment. Consistently, ornithine, the substrate of this enzyme, was found to be decreased. ODC catalyzes the first regulatory step of the synthesis of polyamines, i.e., putrescine, spermidine and spermine. The polyamines are important for the stabilization, repair and expression of DNA, and have a proliferative role [14]. Our results show an increase in the concentration of spermine in active TAO tears, with spermidine not being measurable in our study and putrescine showing only a trend toward increased concentration (*p* = 0.061). It is known that, in rats, thyroid ornithine decarboxylase is strongly stimulated by TSH [15,16], leading to increased levels of polyamines with a proliferative role in goiter and tumor formation [17,18]. As orbital fibroblasts themselves possess their own TSH receptors, playing a pathophysiological role in TAO, the increased synthesis of spermine could also be directly related to the overstimulation by Graves’ autoantibodies and could play a proliferative role at the origin of the increased myogenesis and adipogenesis observed in orbitopathy. Indeed, fibroblasts are known to possess ornithine decarboxylase and polyamines are known to stimulate both myogenesis and adipogenesis [19,20]. In this context, the putrescine/ornithine ratio in tears, the most significant parameter in our study, could be a candidate biomarker of TAO.

In our study, citrulline was found to be lower in the tears of active forms of TAO. Since citrulline may also derivate from ornithine, this later could be preferentially oriented to polyamine production through ODC over-stimulation at the expense of citrulline production. We did not identify a clear relationship in the literature with the other three amino acids whose concentrations were found to be lowered in the tears of active TAO, namely, glycine, histidine and serine.

**Figure 1:**
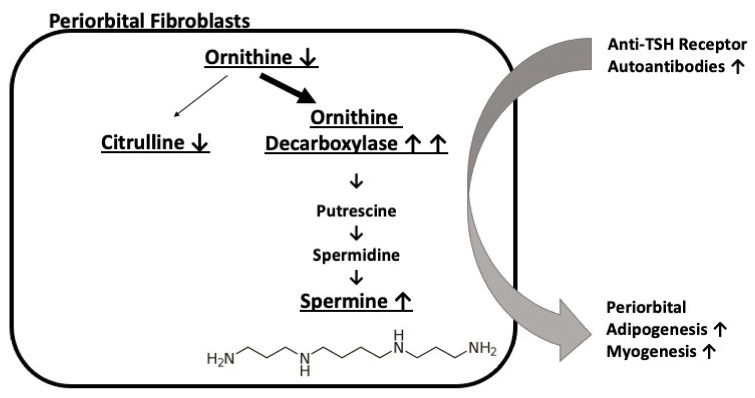
Pathophysiological model suggested by the tear metabolic signature obtained in active versus inactive TAO.

Compared with the metabolomic signatures reported in various situations involving inflammation, at the systemic level or in tears, we did not find any metabolic features reflecting inflammation in our TAO signature. This may be due to the fact that the control group differed little from the TAO group in terms of inflammation. Since this inflammation does not seem to interfere with our signature, this latter is probably quite specific for TAO pathophysiology. However, it is possible that other intercurrent situations, such as the treatments taken by the patients that are often more marked in patients with active orbitopathy, may contribute to this signature. In conclusion, the metabolic signature observed in the tears of active forms of TAO, compared to inactive ones, suggests a pathophysiological mechanism involving the increased spermine synthesis (Figure 1). This metabolic circuit could constitute a local therapeutic target for TAO using ODC inhibitors for their antiproliferative properties. Lastly, the ratio of putrescine to ornithine in tears could be a candidate biomarker of TAO activity.

## Figures and Tables

**Table 1:** Demographic and clinical status of individuals with active and inactive thyroid-associated orbitopathy.

Demographic and Clinical Data	Active (*n* = 21)	Inactive (*n* = 24)	*p*-Values
Demographic data			
Women (%)	76% (16/21)	75% (18/24)	0.93
Mean age (years)	55	51	0.42
Past smoker (%)	42% (5/12)	48% (10/21)	0.74
Active smoker (%)	0% (0/12)	33% (7/21)	0.02
Diabetes (%)	10% (2/21)	4% (1/24)	0.47
Hypertension (%)	19% (4/21)	13% (3/24)	0.55
Hyperlipidemia (%)	5% (1/21)	4% (1/24)	0.92
Graves’ disease features			
TRAb positivity (%)	95% (19/20)	76% (13/17)	0.10
Mean TSH (mUI/L)	3.43	2.84	0.75
Mean fT4 (pmol)	12.35	15.44	0.28
Radiotherapy (%)	14% (3/21)	17% (4/24)	0.83
Corticosteroid (%)	38% (8/21)	37% (9/24)	0.97
Thyroidectomy (%)	19% (4/21)	25% (6/24)	0.63
Duration of dysthyroidism (months)	41	50	0.64
Antithyroid drugs (%)	75% (15/20)	54% (13/24)	0.15
Thyroid hormones (%)	55% (11/20)	71% (17/24)	0.28
Ophthalmological features			
Clinical Activity Score	3.67	0.82	<0.001
Oxford score	0.43	0.08	0.01
Mean visual acuity (log mar)	0.079	0.065	0.68
Mean Schirmer imbibition (mm)	35	32	0.19

**Table 2:** List of metabolites with significant modification of concentrations in tears of patients with active compared to inactive orbitopathy.

Metabolites	*p*-Values	Fold Changes
Spermine	0.0074	1.34
Butyrylcarnitine (C4)	0.0176	1.54
Ornithine	0.0189	0.43
Propionylcarnitine (C3)	0.0232	1.77
Glycine	0.0235	0.45
Serine	0.0278	0.38
Citrulline	0.0281	0.23
Histidine	0.0336	0.45
Ratio		
Putrescine/Ornithine (ornithine decarboxylase activity)	0.0011	3.75

**Table 3:** Demographic and clinical status of individuals with dry eye of undetermined origin.

Demographic and Clinical Data	Controls (*n* = 20)
Women (%)	80%
Mean age (years)	55
Mean Schirmer imbibition (mm)	34.6
Past smoker (%)	36%
Active smoker (%)	27%
Diabetes (%)	25%
Hypertension (%)	25%
Hyperlipidemia (%)	0%
TRAb positivity (%)	NA
Mean TSH (mUI/L)	NA
Mean fT4 (pmol)	NA
Radiotherapy (%)	NA
Corticosteroid (%)	NA
Thyroidectomy (%)	NA
Duration of dysthyroidism (months)	NA
Oxford score	0.15
Mean visual acuity (log mar)	0.1475
Clinical Activity Score	NA

NA—not available.

## Supplementary Materials

The following supporting information can be downloaded at: https://www.mdpi.com/article/10.3390/jcm11020404/s1: Supplementary Table S1. Raw data of metabolites concentration (μM/L) in active and inactive Graves’ ophthalmopathy and *p*-values.
